# Associations of renal sinus fat with metabolic parameters, abdominal visceral adipose tissue, metabolic syndrome, fructose intake, and blood pressure control in obese individuals with hypertension: a cross-sectional study

**DOI:** 10.1017/jns.2024.84

**Published:** 2024-12-16

**Authors:** Paniz Anvarifard, Maryam Anbari, Mohammad Naemi Kermanshahi, Alireza Ostadrahimi, Soghra Aliasgharzadeh, Mohammadreza Ardalan

**Affiliations:** 1 Student Research Committee, School of Nutrition and Food Sciences, Tabriz University of Medical Sciences, Tabriz, Iran; 2 Nutrition Research Center, Tabriz University of Medical Sciences, Tabriz, Iran; 3 Department of Clinical Nutrition, School of Nutrition and Food Sciences, Tabriz University of Medical Sciences, Tabriz, Iran; 4 Kidney Research Center, Tabriz University of Medical Sciences, Tabriz, Iran

**Keywords:** Renal sinus fat, Hypertension, Obesity, Fructose, Visceral adipose tissue, Metabolic syndrome, CKD, chronic kidney disease, MetS, metabolic syndrome, VAT, visceral adipose tissue, SAAT, subcutaneous abdominal adipose tissue, BMI, body mass index, RAAS, renin-angiotensin-aldosterone system, RSF, renal sinus fat, PVAT, perivascular adipose tissue, FBS, fasting blood sugar, PUFT, para- and perirenal ultrasonographic fat thickness, HOMA-IR, homeostasis model assessment of insulin resistance, DBP, diastolic blood pressure, CT, computed tomography, MRI, magnetic resonance imaging, SBP, systolic blood pressure, eGFR, estimated glomerular filtration rate, BIA, bioelectrical impedance analysis, ARB, angiotensin II receptor blockers, ACE, angiotensin converting enzyme, CCB, calcium channel blockers, WHR, waist to hip ratio, IDF, international diabetes federation, TG, triglycerides, HDL-C, high-density lipoprotein cholesterol, FFQ, food frequency questionnaire, FCT, food composition table, USDA, United States Department of Agriculture, TC, total cholesterol, LDL-C, low-density lipoprotein cholesterol, SD, standard deviation, IL-6, interleukin-6, TNF-α, tumour necrosis factor-alpha, ALT, alanine transaminase, AST, aspartate transaminase, VLDL, very-low-density lipoprotein, HFCS, high-fructose corn syrup

## Abstract

Renal sinus fat (RSF) crucially influences metabolic regulation, inflammation, and vascular function. We investigated the association between RSF accumulation, metabolic disorders, and nutritional status in obese individuals with hypertension. A cross-sectional study involved 51 obese hypertensive patients from Salamat Specialized Community Clinic (February–September 2022). Basic and clinical information were collected through interviews. Data included anthropometrics, blood pressure, number of antihypertensive medications, body composition (bioelectrical impedance analysis), dietary intake (semi-quantitative 147-item food frequency questionnaire), and blood samples. Renal sinus fat was measured via ultrasonography. Statistical analyses included Pearson correlation, binary logistic regression, and linear regression. RSF positively correlated with abdominal visceral adipose tissue (VAT) area (*P* = 0.016), systolic blood pressure (SBP) (*P* = 0.004), and diastolic blood pressure (DBP) (*P* = 0.005). A strong trend toward a positive association was observed between antihypertensive medications and RSF (*P* = 0.062). In linear regression, RSF was independently associated with abdominal VAT area, SBP, and DBP after adjusting for confounders. After considering other risk factors, RSF volume relates to prescribed antihypertensive medications, hypertension, and central fat accumulation in obese hypertensive subjects. These findings suggest the need for further investigations into whether RSF promotes metabolic disorders.

## 1. Introduction

Obesity presents a significant global health challenge, with its prevalence having tripled over the past four decades (World Health Organization, 2016). In 2016, more than 1.9 billion adults were classified as overweight, of which over 650 million were categorised as obese, accounting for 39% and 13% of the adult population, respectively (World Health Organization, 2016). Lifestyle changes towards consumption of calorie-dense food and adoption of sedentary lifestyles are two basic forces spreading this malady^(1)^. If current trends persist, the global prevalence of obesity is expected to reach 21% in women and exceed 18% in men by 2025^(2)^. The adverse effects of obesity, particularly abdominal obesity, on various cardiovascular and metabolic conditions such as dyslipidemia, hypertension, diabetes, chronic kidney disease (CKD), cardiovascular disease, and cardiovascular mortality are well-documented^(3)^. Metabolic syndrome (MetS), characterised by the co-occurrence of hyperlipidaemia, insulin resistance, hypertension, and abdominal obesity, is a significant driver of many major diseases and is highly prevalent worldwide^(4,5)^. The incidence of MetS commonly parallels the incidence of obesity and type 2 diabetes mellitus^(6)^. Early diagnosis of MetS is crucial for the identifying high-risk patients who require aggressive lifestyle modifications^(4)^. In recent years, excess abdominal visceral adipose tissue (VAT), also called visceral obesity, rather than total or subcutaneous abdominal adipose tissue (SAAT), has been acknowledged as a primary predictor of metabolic and cardiovascular disease and overall mortality independent of generalised obesity and body mass index (BMI)^(7)^. Visceral adipose tissue is regarded as a form of ‘ectopic fat’, contributing to systemic inflammation, dyslipidemia, insulin resistance, and subsequently, increasing the risk of developing MetS and cardiovascular diseases^(4)^. Ectopic fat can accumulate in various areas of the body, such as the liver, muscle, pericardium, and perivascular area^(8)^. The kidneys, which are surrounded by abdominal VAT, are susceptible to ectopic fat accumulation in the renal sinus^(3)^. The renal sinus is a perirenal region bounded from the hilum of the kidney to the edge of the renal parenchyma where the renal vein, the renal artery, lymphatic vessels, and the ureter enter the kidney^(9)^. Mechanistically, excessive accumulation of fat in the renal sinus can elevate intra-abdominal pressure, compressing low-pressure renal venous structures and leading to renal volume expansion, increased renal interstitial pressure, and activation of the renin-angiotensin-aldosterone system (RAAS)^(9)^. RAAS activation contributes to hypertension, atherosclerosis, insulin resistance, and other obesity-related adverse outcomes^10)^. Renal sinus fat can be easily measured by computed tomography (CT), magnetic resonance imaging (MRI), and ultrasound^(4)^. The renal sinus fat (RSF) is similar to perivascular adipose tissue (PVAT) in terms of its characteristics. Perivascular adipose tissue is a type of active endocrine tissue that plays a crucial role in regulating inflammation, vascular function, and metabolism^(11,12)^. These characteristics suggest a potential role for RSF in MetS regulation. Recent studies have linked RSF to MetS components such as central obesity, hypertension, insulin resistance, and dyslipidemia. Chughtai HL. et al. demonstrated that higher RSF volume was independently associated with stage II hypertension, an increased number of medications required for hypertension management, abdominal fat, and hyperlipidaemia in individuals at risk for cardiovascular events. However, their study found no significant difference in RSF volume between patients with and without diabetes, nor was RSF associated with fasting blood sugar (FBS) or BMI^(10)^. Similarly, a cross-sectional study involving individuals with normal glucose levels, prediabetes, and diabetes revealed that RSF volume increased significantly in prediabetic subjects and was strongly associated with VAT and hypertension^(13)^. De Pergola G. et al. found a positive association between para- and perirenal ultrasonographic fat thickness (PUFT) and waist circumference, insulin levels, homeostasis model assessment of insulin resistance (HOMA-IR), and mean 24-hour diastolic blood pressure (DBP) in overweight and obese individuals. However, no significant correlation was observed between PUFT and BMI or FBS^(14)^. Another cross-sectional study identified adipose tissue deposition, particularly in the left renal sinus, as being related to VAT levels; however, reductions in VAT were not mirrored by decreases in RSF accumulation^(15)^. Additionally, Guo XL. et al. showed that perirenal fat thickness was significantly associated with MetS^(4)^.

Modern communities, mainly those with high obesity rates, are characterised by high intake of fructose^(16)^. Despite the rationale that dietary fructose and fructose-sweetened beverage consumption can disturb several functions in adipocytes and increase VAT, to date, no studies have yet explored the association between RSF and fructose intake^(7,16)^.

Based on previous anatomical and cross-sectional studies, we hypothesised that increased RSF is associated with a higher risk of developing MetS. Additionally, high-fructose consumption is expected to contribute to RSF expansion. Therefore, we designed this cross-sectional study to examine the associations between RSF, metabolic parameters, abdominal VAT, MetS, fructose intake, and blood pressure control in obese individuals with hypertension.

## 2. Materials and methods

### 2.1. Study design

In the current cross-sectional study, obese patients with hypertension were consecutively enrolled using the convenience sampling method from Salamat Specialized Community Clinic, Tabriz, Iran, from February 2022 to September 2022. The study was approved by the Medical Ethics Committee of Tabriz University of Medical Sciences (approval number: IR.TBZMED.REC.1399.1173) and was carried out according to the latest version of the Declaration of Helsinki. All participating patients provided written consent prior to their involvement in the study.

### 2.2. Study population

The study included hypertensive patients aged 20-75 years with BMI over 30 kg/ 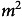 . Hypertension was characterised by a systolic blood pressure (SBP) equal to or exceeding 130 mmHg, DBP equal to or exceeding 80 mmHg, or the utilisation of antihypertensive medication^(17)^. Patients with renal abnormalities (such as a difference in kidney length between the right and left side of more than 1.5 cm, solitary kidney or multiple kidneys, polycystic kidney, pelvic kidney, glomerulonephritis, hydronephrosis, renal artery stenosis, or congenital renal anomalies), renal transplant, history of renal surgery, estimated glomerular filtration rate (eGFR) < 45 ml/min/1.73 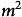 , liver cirrhosis, active cancer, and those who were currently pregnant or breastfeeding were excluded from the study. Additionally, subjects with an implantable cardioverter defibrillator or pacemaker were excluded due to the conditions required for performing bioelectrical impedance analysis (BIA). The sample size was determined using PASS software (version 15.0.5) based on the results of a previous related study^(14)^. Pearson’s correlation test was selected to calculate the appropriate sample size, considering the association between PUFT and waist circumference^(14)^. The minimum required sample size was determined to be 49 subjects, with an alpha level of G0.05, power level of 80%, and a Pearson’s correlation coefficient of 0.39. On this basis and considering the inclusion and exclusion criteria, a total of 51 subjects (39.2% male, 60.8% female) out of the initially screened 202 subjects were enrolled in the current study.

### 2.3. Socio-demographic, blood pressure, anthropometric, and body composition assessments

We gathered socio-demographic data including gender, age, educational background, occupation, marital status, smoking habits, and medical history through structured interviews. Blood pressure measurements were taken using a mercury sphygmomanometer (ALPK2, Japan) twice after a 30-minute rest in a seated position. The mean of the two readings was reported as the final result. We adopted the American College of Cardiology/American Heart Association (ACC/AHA) hypertension guidelines, whereby stage I hypertension was identified as having a SBP ranging from 130 to 139 mmHg or DBP ranging from 80 to 89 mmHg, and stage II hypertension was defined as having an SBP of 140 mmHg or higher or a DBP of 90 mmHg or higher. Antihypertensive agents were classified as angiotensin II receptor blockers (ARB), angiotensin-converting enzyme (ACE) inhibitors, calcium channel blockers (CCB), beta-blockers, alpha-blockers, combined alpha and beta-blockers*, alpha-2* receptor *agonists,* diuretics, and direct vasodilators. Patients were categorised based on the number of antihypertensive agents they were receiving (1, 2, 3 or more). Body weight and height were measured with participants in a straight standing position, without shoes, and with light clothing using a digital Seca scale (Seca 22089, Hamburg, Germany) and a portable stadiometer (Seca, Hamburg, Germany) with an accuracy of approximately 100 g and 0.5 cm, respectively. The body mass index was computed by dividing the body weight by the square of the height (kg/ 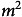 ). Waist and hip circumferences were measured using a nonstretchable tape to the nearest 0.1 cm at the narrowest area of the abdomen (midpoint of the lowest rib and iliac crest) and widest area of the hips (greatest protuberance of the buttocks), respectively. Waist-to-hip ratio (WHR) was calculated by dividing the waist measurement by the hip measurement. Body composition, including VAT ( 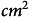 ), was evaluated using BIA technology (Tanita BC-420MA; Tokyo, Japan), following standard procedures^(18)^. Participants were instructed not to engage in strenuous physical activity and to avoid consuming alcohol or caffeine the day before the BIA. They were also asked to be well hydrated but to stop drinking water an hour before the analysis. The analysis was performed after a 12-hour fasting period and with an empty bladder. All study subjects received a low-calorie diet for weight management and were encouraged to enhance their physical activity.

### 2.4. Definition of MetS

Metabolic syndrome was defined according to the 2006 international diabetes federation (IDF) parameters as abdominal obesity (waist circumference ≥ 94 in men, ≥ 80 cm in women), along with any two of the following criteria: (1) SBP of at least 130 mmHg or DBP of at least 85 mmHg, or the use of antihypertensive medications; (2) FBS ≥ 100 mg/dL or previously diagnosed diabetes mellitus with treatment; (3) fasting triglycerides (TG) ≥ 150 mg/dL or ongoing treatment for elevated TG; (4) high-density lipoprotein cholesterol (HDL-C) level < 40 mg/dL in men, < 50 mg/dL in women^(19)^. We divided participants into the MetS- and MetS + groups.

### 2.5. Assessment of dietary intake

Usual dietary intakes of the study subjects were assessed using a semi-quantitative 147-item food frequency questionnaire (FFQ) that had been previously evaluated for reliability and validity^(20,21)^. All questionnaires were administered through individual interviews conducted by qualified dietitians. The FFQ included a list of food items with standard serving sizes mostly consumed by Iranians. Participants were asked to report the frequency and amount of consumption of each item based on serving size during the last year, on a daily, weekly, monthly, or yearly basis. The portion size of consumed foods was converted to daily intakes (grams) using household measures. Daily intake of energy and each nutrient, as well as total fructose, was determined using the Iranian food composition table (FCT)^(22)^ and United States Department of Agriculture (USDA) food composition data^(23)^.

### 2.6. Measurement of RSF

To measure RSF, we followed the same method as previously described by others^(14)^. We used a duplex Doppler ultrasound apparatus (Acuson Sequoia 512 ultrasound system, Siemens, USA) to conduct the ultrasound examinations. The patients were positioned supine, and the probe was placed perpendicular to the skin on the lateral side of the abdomen. Longitudinal scanning was performed, and the optimal position, where the surface of the kidney was almost parallel to the skin, was found by slowly moving the probe laterally. Minimal pressure was applied to the probe to avoid compressing the fat layers. The ultrasound volume of RSF from the inner side of the abdominal musculature to the surface of the kidney was measured. The average of the maximal volumes on both sides was taken as the RSF. The correlation between RSF values measured on both sides was 0.849 (*P* < 0.001). RSF was measured three times. The intraoperator coefficient of variation was 4.6 %. The sonographer conducting the ultrasound examinations was blinded to all other aspects of the study.

### 2.7. Biochemical assessments

A fasting blood sample was obtained from each participant and then centrifuged to separate serum. Serum TG, total cholesterol (TC), HDL-C, and FBS were measured using commercial kits (Mancompany, Tehran, Iran) in accordance with the manufacturer’s instructions. All biochemical tests were performed on fresh blood samples. The concentration of low-density lipoprotein cholesterol (LDL-C) was calculated using the Friedewald formula^(24,25)^.

### 2.8. Statistical analysis

The data were first examined for normal distribution by using the Shapiro-Wilk test. Results were expressed as mean ± standard deviation (SD) for normally distributed continuous values, median (interquartile range 25-75 percentile) for data with skewed distribution, or frequency (percentage) for qualitative variables. We compared two groups using independent samples t-test for normally distributed continuous variables and Mann–Whitney U test for non-normally distributed variables. Pearson or Spearman correlation coefficients, as appropriate, were used to evaluate univariate correlations between RSF and all investigated parameters. Linear regression analyses were used to assess the significance of covariate-adjusted cross-sectional relation of RSF (dependent variables) with VAT, SBP, and DBP. Furthermore, to test the independent relationship between MetS (dependent variables) and RSF, we constructed binary logistic regression analysis. Data are expressed as unstandardised (*B*) regression coefficient. All analyses were conducted using IBM SPSS Statistics software, version 25 (SPSS Inc., and Chicago, IL, USA); *P* < 0.05 was considered statistically significant.

## 3. Results

The general, metabolic, anthropometric, and dietary parameters of the study participants are described in Table 1. The mean age of the 51 obese patients with hypertension was 53.39 ± 9.84, ranging from 29 to 70 years old. The mean value of RSF in the study sample was 24.24 ± 11.10. The prevalence of MetS was 84.3%. Patients with and without diabetes had similar amounts of RSF (*P* = 0.14). Table 2 displays the correlations of RSF with all investigated parameters in a sample of 51 study participants. RSF was significantly and positively associated with abdominal VAT area (*r* = *0.335, P* = 0.016), but not associated with waist circumference (*P* = 0.657) and BMI (*P* = 0.554). Male patients had significantly greater amounts of the VAT area (167.75 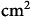 versus 121.84 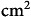 ; *P* = 0.017), waist circumference (112.70 cm versus 107.80 cm; *P* = 0.056), and WHR (0.97 versus 0.90; *P* < 0.001) compared with female patients. On the contrary, the BMI level was significantly higher in female than male subjects (35.67 kg/ 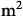 versus 32.16 kg/ 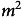 ; *P* <0.001). As the number of antihypertensive medications taken by the participants increased, there was a strong trend toward a positive correlation (*r* = 0.264, *P* = 0.062) with RSF volume, indicating an increase in RSF volume. Additionally, the correlations of RSF with SBP (*r =*0.395, *P* = 0.004) and DBP (*r =* 0.391, *P* = 0.005) were statistically and positively significant. However, there were no significant differences in RSF volume between participants with stage I hypertension and those with stage II hypertension (*P* = 0.484). Neither lipid profile measures, including TG, TC, HDL-C, and LDL-C nor FBS showed a significant correlation with RSF (*P* = 0.592, *P* = 0.829, *P* = 0.383, *P* = 0.673, *P* = 0.491, respectively). Moreover, there were no significant correlations found between RSF and total daily fructose intake (*P* = 0.869) or total daily energy intake (*P* = 0.737). It should be noted that there was a significant positive correlation between fructose and energy intake (*r =* 0.651, *P* < 0.001), so the daily intake of fructose was adjusted for energy intake using the residual method^(26)^, but still, no significant association with RSF was observed (*P* = 0.769). Patients with stage II hypertension were found to have a higher level of fructose consumption compared to those with stage I hypertension (*P* = 0.041). However, this significance disappeared after adjusting for energy intake (*P* = 0.297).

**Table 1.** Characteristics of the study population

Clinical characteristics	All (*n* = 51)
Age, years	53.39 ± 9.84
Male, *n* (%)	20 (39.2)
Diabetes, *n* (%)	13 (25.5)
MetS, *n* (%)	43 (84.3)
Weight, kg	90.08 ± 12.00
Height, cm	162.20 ± 10.44
BMI, kg/ 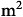	34.30 ± 4.03
Waist circumference, cm	109.72 ± 8.96
SBP, mmHg	135 (125.00–145.00)
DBP, mmHg	90 (85.00–100.00)
FBS, mg/dl	101.00 (93.75–119.25)
TG, mg/dl	155 (117.50–228.50)
TC, mg/dl	173.18 ± 47.90
HDL-C, mg/dl	42.50 ± 9.79
LDL-C, mg/dl	106.52 ± 40.44
RSF, cc	24.24 ± 11.10
VAT area, 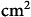	139.84 ±67.92
Total daily energy intake, kcal	2541.71 (2030.63–3325.95)
Total daily fructose intake, g	21.32 (16.30–32.10)

The data are presented as means ± standard deviation (SD), medians (interquartile range), or frequencies. MetS, metabolic syndrome; BMI, body mass index; SBP, systolic blood pressure; DBP, diastolic blood pressure; FBS, fasting blood sugar; TG, triglycerides; TC, total cholesterol; HDL-C, high-density lipoprotein cholesterol; LDL-C, low-density lipoprotein cholesterol; RSF, renal sinus fat; VAT, visceral adipose tissue.

**Table 2.** Correlations between RSF (cc) and all other investigated parameters in 51 subjects under study

Variables	r	*P*-value
BMI, kg/ 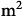	−0.085	0.554^b^
Waist circumference, cm	0.064	0.657^b^
VAT area, 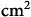	0.335	**0.016** ^**b**^
SBP, mmHg	0.395	**0.004** ^a^
DBP, mmHg	0.391	**0.005** ^a^
Number of antihypertensive agents	0.264	**0.062** ^a^
FBS, mg/dl	0.104	0.491^a^
TG, mg/dl	0.078	0.592^a^
TC, mg/dl	−0.032	0.829^b^
HDL-Cholesterol, mg/dl	−0.150	0.383^b^
LDL-Cholesterol, mg/dl	−0.073	0.673^b^
Total daily energy intake, kcal	−0.048	0.737^a^
Total daily fructose intake, g	−0.024	0.869^a^
Energy-adjusted fructose intake, g	−0.042	0.769^a^

a Data show the Spearman correlation coefficient.

b Data show the Pearson correlation coefficient.

RSF, renal sinus fat; BMI, body mass index; VAT, visceral adipose tissue; SBP, systolic blood pressure; DBP, diastolic blood pressure; FBS, fasting blood sugar; TG, triglycerides; TC, total cholesterol; HDL, high-density lipoprotein; LDL, low-density lipoprotein.

The association between MetS and RSF was investigated by binary logistic regression analysis (Table 3). Metabolic syndrome (dependent variable) showed no association with RSF (OR = 1.029, 95% CI, 0.936-1.131; *P* = 0.560).

**Table 3.** The prediction power of MetS by RSF based on binary logistic regression analysis

Dependent variable	Unstandardised *β*	OR	*P*-value	95% CI
MetS	0.028	1.029	0.560	0.936–1.131

MetS, metabolic syndrome; RSF, renal sinus fat; OR, odds ratio; CI, confidence interval.

**Figure 1.**
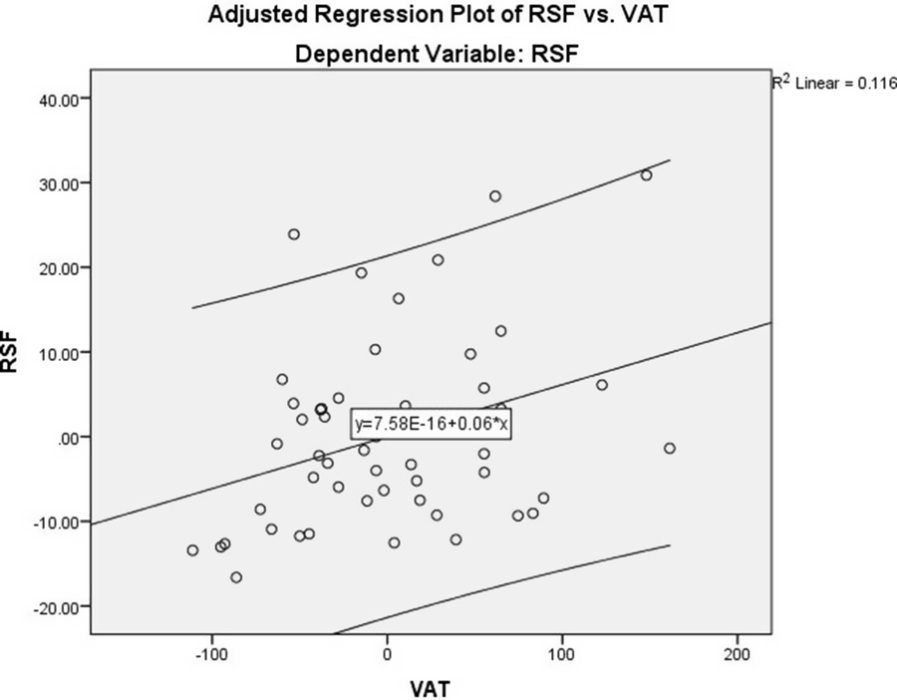
Adjusted regression plot showing the relationship between RSF and VAT, adjusted for waist circumference. The regression line demonstrates a significant positive association.

**Figure 2.**
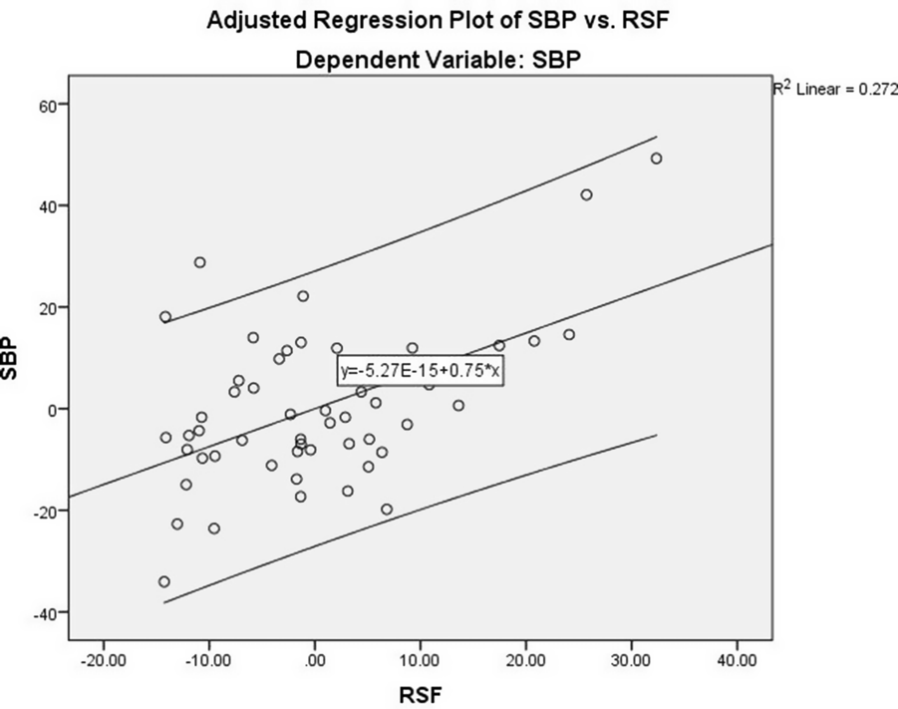
Adjusted regression plot illustrating the independent association between RSF and SBP after controlling for age and gender. The results indicate that RSF is a significant predictor of SBP in hypertensive obese individuals.

**Figure 3.**
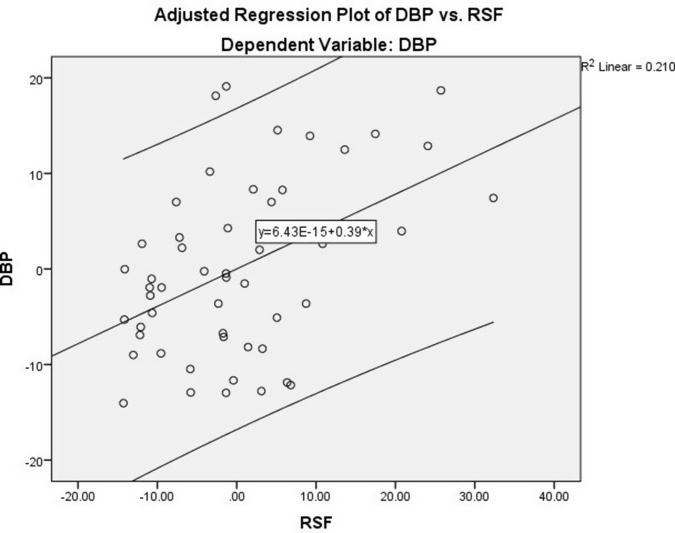
Adjusted regression plot depicting the correlation between RSF and DBP. The relationship is adjusted for relevant confounders, showing a positive association between RSF and DBP.

To further confirm the associations of VAT, SBP, and DBP with RSF, we performed linear regression analyses. Using the RSF as a dependent variable and waist circumference as a covariate (Table 4), the results showed that VAT was a significant and independent predictor for RSF (*B* = 0.061, 95% CI, 0.012–0.111; *P* = 0.015) after adjusting for confounding factor (Model 1) (Figure 1). Moreover, considering SBP as an outcome variable and independent variables including RSF, age, and gender, the results showed that RSF was independently correlated with SBP (Table 5) (Figure 2). As shown in Table 5, linear regression analysis also confirmed that the association of DBP (dependent variable) and RSF was independent of other variables added to the model (age and gender) (Figure 3).

**Table 4.** The prediction power of RSF by VAT based on linear regression analysis

Dependent variable		Unstandardised *B* (95% CI)	*P*-value
RSF	Crude	0.055 (0.011–0.099)	0.016
	Model 1	0.061 (0.012–0.111)	0.015

Model 1: Adjusted for waist circumference.

RSF, renal sinus fat; VAT, visceral adipose tissue; CI, confidence interval.

**Table 5.** The prediction power of SBP and DBP by RSF based on linear regression analyses

Dependent variable		Unstandardised *B* (95% CI)	*P*-value
SBP	Crude	0.769 (0.407–1.132)	<0.001
	Model 1	0.746 (0.387–1.104)	<0.001
DBP	Crude	0.418 (0.204–0.633)	<0.001
	Model 1	0.391 (0.169–0.614)	0.001

Model 1: Adjusted for age and gender.

SBP, systolic blood pressure; DBP, diastolic blood pressure; RSF, renal sinus fat; CI, confidence interval.

## 4. Discussion

The aim of this study is to explore the potential link between RSF and various anthropometric and metabolic parameters, MetS, and fructose consumption among obese individuals with hypertension. The findings of this research reveal a substantial positive correlation between RSF and abdominal VAT area. Additionally, we observed a positive correlation between RSF and both SBP and DBP. However, we did not identify any significant association between RSF and waist circumference or BMI. Furthermore, there was no significant relationship between RSF and fructose intake or MetS. Although a marginally significant positive correlation was found between the volume of RSF and the number of antihypertensive medications taken, no significant correlation was observed between RSF and lipid profile measures, FBS, or daily energy intake.

Obesity is a significant global public health concern and is associated with several chronic illnesses^(27)^. The prevalence of obesity is increasing worldwide^(28)^, and studies have shown that obesity is directly related to increased blood pressure and the risk of MetS^(29,30)^. Furthermore, the intake of fructose has been linked to an increased risk of obesity and metabolic diseases^(31,32)^. Therefore, treatments aimed at reducing the volume of RSF as a major predictor of metabolic diseases and hypertension are crucial for the management of obesity.

### 4.1. Renal sinus fat and anthropometric indices

The results of this study support the hypothesis that VAT plays a role in the accumulation of RSF. Linear regression analysis showed that VAT was a significant and independent predictor for RSF, even after adjusting for confounding factor. These findings are consistent with previous research demonstrating that following bariatric surgery, RSF was reduced along with other markers of adiposity in obese patients^(33)^. Additionally, a cohort study found that the accumulation of central fat in healthy overweight and obese individuals is associated with an increase in pararenal, perirenal, and epicardial fat^(34)^. The results of another study showed that perirenal fat thickness was significantly correlated with metabolic risk factors like BMI and waist circumference^(35)^. Obesity is characterised by excessive accumulation of adipose tissue, including VAT, which releases various bioactive molecules called adipokines^(36,37)^. Pro-inflammatory cytokines such as interleukin-6 (IL-6), tumour necrosis factor-alpha (TNF-α), and adiponectin play an important role in the development of chronic low-grade inflammation, which is a hallmark of obesity^(38,39)^. The chronic inflammatory state created by obesity can lead to the recruitment and activation of immune cells, such as macrophages, in the renal sinus. These activated immune cells release additional pro-inflammatory cytokines, perpetuating the inflammatory response in the renal sinus^(40)^.

### 4.2. Renal sinus fat and blood pressure

The results of this study suggest that RSF may be associated with blood pressure, as there was a significant correlation between RSF and SBP or DBP. However, there were no significant differences in RSF volume between participants with stage I hypertension and those with stage II hypertension. Additionally, there was a strong trend toward a positive correlation between the number of antihypertensive medications taken and RSF volume, suggesting that an increase in RSF volume may be associated with a higher number of antihypertensive medications. Several studies have demonstrated a significant association between RSF and hypertension, as well as the number of prescribed antihypertensive medications and renal size^(13,33)^. Furthermore, RSF has been shown to be associated with SBP, DBP, and mean arterial pressure regardless of visceral adiposity and BMI^(3,41)^. This may be due to the compression of renal structures, leading to increased renal interstitial pressure, activation of the RAAS, and retention of sodium^(42,43)^. Consistent with our study, the Framingham Heart Study found a positive association between RSF and hypertension, SBP, and DBP^(3)^. The excessive accumulation of fat in the renal sinus may lead to an increase in intra-abdominal pressure and compression of the low-pressure renal veins, causing changes in kidney function through the activation of the RAAS. Therefore, the expansion of fat in the renal sinus may contribute to the worsening of hypertension and renal dysfunction in individuals with obesity^(41,42)^.

### 4.3. Renal sinus fat and MetS

Lipid profile measures (TG, TC, HDL-C, LDL-C) and FBS did not show a significant correlation with RSF. Binary logistic regression analysis showed no association between MetS and RSF.

The significance of RSF in examining cardiovascular risk factors in MetS has gained attention. Perivascular adipose tissue plays a crucial role in linking obesity, liver function, insulin resistance, and both macro- and microangiopathy across multiple organs^(44,45)^. Recent studies on the relationship between RSF and MetS have been challenging and contradictory. Contrary to the findings of the present study, the results of Notohamiprodjo M. et al trial indicate a significant increase in RSF in individuals with prediabetes to healthy subjects and RSF as a PVAT acts as a potential imaging biomarker as an important predictor of metabolic diseases^(13)^. In another retrospective study, patients with MetS had greater perirenal fat thickness, HOMA-IR, alanine transaminase (ALT), and aspartate transaminase (AST) than those without MetS^(46)^. But consistent with our findings, the results of another study have shown no association between insulin sensitivity with VAT, intra-hepatic lipid, intra-pancreatic lipid, and intra-myocellular lipids in black West African men^(47)^.

It appears that various factors such as age, sex, and ethnicity are involved in the relationship between RSF and MetS. Recent studies indicate that the quality of adipose tissue in different anatomic regions and the effect of renal sinus adipose tissue quality on renal dysfunction are effective in developing MetS^(8)^. Although we did not find an association between RSF and MetS, it is possible that different results would have been obtained with a larger sample size. Nevertheless, these findings should be further investigated in a larger population.

### 4.4. Renal sinus fat and fructose intake

Our study showed that daily fructose intake did not have a significant correlation with RSF. To the best of our knowledge, no study has examined the direct relationship between fructose intake and RSF. Recent studies have focused on the association between fructose intake and lipogenesis, as well as chronic diseases such as diabetes, hypertension, and obesity^(16,48)^. Fructose is primarily metabolised in the liver, where it undergoes phosphorylation by fructokinase, leading to the formation of fructose-1-phosphate. This process bypasses the main regulatory step of glycolysis, resulting in uncontrolled glycolytic flux. Excessive fructose metabolism leads to increased production of acetyl-CoA, which promotes de novo lipogenesis and TG synthesis^(49,50)^. Elevated TG levels can subsequently contribute to ectopic fat deposition, including the renal sinus^(51)^.

A clinical trial showed that a 7-day high-fructose diet increased fasting very-low-density lipoprotein (VLDL) triacylglycerols and ectopic lipid deposition in the liver and muscle, and decreased hepatic insulin sensitivity in healthy subjects with a family history of type 2 diabetes^(48)^. However, in line with our study, the results of Bravo S. et al indicated that normal consumption of fructose as part of a typical diet in commonly consumed sweeteners, such as sucrose or high-fructose corn syrup (HFCS), does not promote ectopic fat storage in the liver or muscles^(52)^.

Although we did not find a correlation between fructose intake and RSF, it is possible that fructose metabolism disrupts lipid homeostasis, leading to ectopic fat deposition within the renal sinus. This could be due to the misclassification of study participants resulting from the use of FFQ. Further studies are needed to explore therapeutic interventions targeting fructose metabolism and lipogenesis to mitigate the detrimental effects on RSF volume and metabolic disorders.

### 4.5. Strengths and limitations of this study

This study has several strengths worth highlighting. First, we had access to comprehensive information on both dietary and non-dietary factors, which allowed us to control for a broad range of potential confounders and obtain more robust independent associations. Second, the use of validated questionnaires for data collection enhances the reliability and accuracy of our findings. However, there are also several limitations to consider. Firstly, due to the cross-sectional nature of this study, causality cannot be established for the observed associations. Secondly, although we controlled for most lifestyle factors and diet quality, residual or unmeasured confounding may still influence the results. Furthermore, while the sample size was calculated using an appropriate formula, larger sample sizes are needed to confirm these findings. Additionally, as with many studies in nutritional epidemiology, there is a potential for participant misclassification due to the use of FFQ. Lastly, the lack of longitudinal follow-up limits our ability to assess the progression of RSF accumulation over time and its long-term effects on the variables measured.

### 4.6. Conclusion

Overall, these findings suggest that RSF is positively associated with abdominal VAT area, SBP, DBP, and antihypertensive medication use. However, no significant associations were observed between RSF and other anthropometric, metabolic, or dietary parameters, including MetS. These results highlight the potential of VAT as a contributor to RSF accumulation, emphasising the importance of managing VAT in clinical strategies aimed at reducing RSF and improving blood pressure control. Identifying individuals with excessive VAT could help tailor interventions to limit RSF accumulation and better manage hypertension. Further, longitudinal studies are needed to establish causality and elucidate the underlying mechanisms linking RSF accumulation to metabolic disorders and nutritional status, ultimately guiding more effective prevention and treatment strategies.

## Supporting information

Anvarifard et al. supplementary material Anvarifard et al. supplementary material

## Data Availability

Data described in the manuscript and analytic code will be made available from the corresponding author upon reasonable request.

## Supplementary material

The supplementary material for this article can be found at https://doi.org/10.1017/jns.2024.84.
