# Supplementary material for: Associations of renal sinus fat with metabolic parameters, abdominal visceral adipose tissue, metabolic syndrome, fructose intake, and blood pressure control in obese individuals with hypertension: a cross-sectional study
Source: J Nutr Sci. 2024 Dec 16;13:e94. doi: 10.1017/jns.2024.84 (PMC11658955; doi:10.1017/jns.2024.84)
Supplement: Anvarifard et al. supplementary material [file S2048679024000843sup001.docx]

- Renal Sinus Fat (RSF) is crucial for treating obese hypertension
- RSF correlates with antihypertensive medications, severity, and central fat
- Non-invasive methods (e.g., ultrasonography) assess RSF
- RSF evaluation offers insights for tailored treatment
- Potential for discovering new approaches in metabolic disorder management
